# The Minimum Quality Criteria Set (QI-MQCS) for critical appraisal: advancing the science of quality improvement

**DOI:** 10.1186/1748-5908-10-S1-A19

**Published:** 2015-08-20

**Authors:** Lisa V Rubenstein, Susanne Hempel, Jodi L Liu, Margie J Danz, Robbie Foy, Yee-Wei Lim, Aneesa Motala, Paul G Shekelle

**Affiliations:** 1 Department of Medicine, VA Greater Los Angeles, North Hills, CA 91343, USA; 2 RAND Health, RAND Corporation, Santa Monica, CA 90407, USA; 3 Department of Medicine, University of California, Los Angeles, Los Angeles, CA 91343, USA; 4 Institute of Health Science, University of Leeds, Leeds, LS2 9LJ, UK; 5 Saw Swee Hock School of Public Health, National University of Singapore, 117549, Singapore

## Objective

Effective learning across related scientific investigations through evidence synthesis is critical to promoting evidence-based approaches to healthcare. Synthesis of findings from quality improvement intervention (QII) publications, however, poses challenges. We aimed to develop a critical appraisal instrument (the Minimum Quality Criteria Set or QI-MQCS) to promote identification, dissemination and implementation of findings from high quality QII evaluations.

## Methods

We convened a 9 person expert panel to guide QII evidence synthesis methods development through a one year iterative telephone, survey and in-person panel process. We developed and empirically tested electronic search and screening methods for identifying QII publications, and a critical appraisal instrument. Finally, we iteratively tested and improved QI-MQCS psychometric properties based on review of 54 electronically searched and systematically screened QII articles.

## Results

Panelists agreed QI-MQCS should focus on QII specific domains, not evaluation design criteria. The 16 QI-MQCS domains address Organizational Motivation, Intervention Rationale, Intervention Description, Organizational Characteristics, Implementation, Study Design, Comparator Description, Data Sources, Timing, Adherence / Fidelity, Health Outcomes, Organizational Readiness, Penetration / Reach, Sustainability, Spread, and Limitations. The median inter-rater agreement for QI-MQCS items was kappa 0.57 (83% agreement). Items discriminated between studies in terms of quality (median criteria met 67%). Internal consistency measures indicated coherence without excessive conceptual overlap (Cronbach's alpha = 0.60, absolute mean inter-item correlation = 0.19). The critical appraisal instrument is accompanied by a user manual detailing What to consider, Where to look, and How to rate.

## Conclusions

The QI-MQCS had acceptable psychometric properties for critical appraisal, and can support systematic review of diverse QII evaluations. It is a ready-to-use critical appraisal tool accompanied by a user manual and empirically tested forms and methods.

